# Effects of defined gut microbial ecosystem components on virulence determinants of *Clostridioides difficile*

**DOI:** 10.1038/s41598-018-37547-x

**Published:** 2019-01-29

**Authors:** Christian Carlucci, Carys S. Jones, Kaitlyn Oliphant, Sandi Yen, Michelle Daigneault, Charley Carriero, Avery Robinson, Elaine O. Petrof, J. Scott Weese, Emma Allen-Vercoe

**Affiliations:** 1 Department of Molecular and Cellular Biology, University of Guelph, Guelph, Ontario Canada; 2 Division of Infectious Diseases/Gastrointestinal Diseases Research Unit, Department of Medicine, Queen’s University, Kingston, Ontario Canada; 3 Department of Pathobiology, Ontario Veterinary College, University of Guelph, Guelph, Ontario Canada

## Abstract

Many cases of *Clostridioides difficile* infection (CDI) are poorly responsive to standard antibiotic treatment strategies, and often patients suffer from recurrent infections characterized by severe diarrhea. Our group previously reported the successful cure of two patients with recurrent CDI using a standardized stool-derived microbial ecosystem therapeutic (MET-1). Using an *in vitro* model of the distal gut to support bacterial communities, we characterized the metabolite profiles of two defined microbial ecosystems derived from healthy donor stool (DEC58, and a subset community, MET-1), as well as an ecosystem representative of a dysbiotic state (ciprofloxacin-treated DEC58). The growth and virulence determinants of two *C. difficile* strains were then assessed in response to components derived from the ecosystems. CD186 (ribotype 027) and CD973 (ribotype 078) growth was decreased upon treatment with DEC58 metabolites compared to ciprofloxacin-treated DEC58 metabolites. Furthermore, CD186 TcdA and TcdB secretion was increased following treatment with ciprofloxacin-treated DEC58 spent medium compared to DEC58 spent medium alone. The net metabolic output of *C. difficile* was also modulated in response to spent media from defined microbial ecosystems, although several metabolite levels were divergent across the two strains examined. Further investigation of these antagonistic properties will guide the development of microbiota-based therapeutics for CDI.

## Introduction

*Clostridioides difficile* is an anaerobic, exotoxin-producing, sporulating bacterial pathogen that is one of the most common causes of antibiotic-associated diarrhea^1,2^. Many epidemic-associated *C. difficile* strains secrete a combination of TcdA, TcdB, and CDT toxins into the colonic lumen, where they may be taken up by colonocytes, mediating the disease symptoms of *C. difficile* infection (CDI)^3^. CDI severity can range from mild, self-limiting diarrhea to severe gastrointestinal complications that result in significant patient morbidity and extensive healthcare costs^1^.

Through a variety of proposed mechanisms, it is thought that the gut microbiome facilitates colonization resistance against *C. difficile*, suppressing its expansion in the colon^4–7^. However, disturbances to host-gut microbiota homeostasis, predominantly through the use of antibiotic treatments given for unrelated clinical indications, can lead to CDI^7–11^. Standard treatment practices for CDI include the use of additional antibiotics active against the vegetative form of *C. difficile*, which further disrupt the gut microbiota and fail to eradicate *C. difficile* endospores (spores), which are resistant to antibiotics. Endospores then germinate, often leading to recurrence of disease^1,12^. Recurrent CDI (rCDI) occurs in up to 30% of patients being treated for a first episode CDI, and rCDI rates are on the rise^13,14^. As traditional antibiotic treatment regimens are not as effective against rCDI, there has been intense interest in the development of microbiota-based therapeutics for this indication.

Among novel therapeutics, fecal microbiota transplantation (FMT) is a highly effective intervention for rCDI^14–19^, now recommended in many countries for cases of multiple rCDI where standard antibiotic treatments have failed^20,21^. FMT has been shown to restore compositional and functional microbial ecosystem imbalances present in the gut microbiome of rCDI patients. Taxonomic changes in the gut microbial composition of CDI patients following FMT have been well characterized. In general, an expansion of bacterial species within Firmicutes (specifically within the families Lachnospiraceae, Ruminococcaceae and Clostridiaceae) and Bacteroidetes (specifically within the families Bacteroidaceae, Rikenellaceae and Porphyromonadaceae), with concomitant decreases in Proteobacteria (in particular members of the family Enterobacteriaceae) has been observed in comparison to pre-FMT patient samples^17,18,22–28^. Although not fully understood, it is thought that FMT may mediate the restoration of metabolic imbalances of short-chain fatty acids (SCFA)^8^, sialic acid^6^, succinate^5^, and bile acids^4,29,30^ associated with CDI, in turn affecting both the host immune response^25^ and expansion of *C. difficile*. While the reconstitution of metabolic imbalances through FMT has been shown to influence *C. difficile* growth and germination^31^, the effects on sporulation and toxin production are less characterized. This is an important knowledge gap considering the importance of these virulence determinants in the pathophysiology of CDI.

Although FMT is highly effective for rCDI, the use of stool as a therapeutic presents many logistical, regulatory, and safety challenges. To address these issues, our group previously developed a defined microbial ecosystem therapeutic (MET-1), derived from the stool of a healthy fecal donor, which was used to cure two patients with rCDI^32^. Although the precise mechanism through which health is restored in rCDI patients after MET-1 treatment is not fully understood, preliminary evidence of host-associated protective effects and direct antagonistic mechanisms against *C. difficile* TcdA have been described^33,34^.

This study aimed to examine alterations in virulence determinants of two clinically relevant, epidemic-associated *C. difficile* strains, CD186 (ribotype 027/NAP1/toxinotype III) and CD973 (ribotype 078, NAP7/toxinotype V) in response to defined microbial ecosystem components.

## Results

### Development and characterization of defined microbial ecosystems

Bacterial isolates derived from the stool of a healthy fecal donor were used to develop defined gut microbial ecosystems, as previously described^32^. A defined experimental community of 58 bacterial isolates (DEC58) and a subset community of DEC58 species comprising 33 strains, MET-1 (Supplementary Table S1), were seeded into bioreactors set to model the conditions of the human distal gut. A third ecosystem representative of a dysbiotic gut microbial community was formulated through the application of a physiologically relevant dose of ciprofloxacin, a clinically important broad-spectrum antibiotic associated with CDI, to bioreactor-cultured DEC58. Bioreactor vessels were allowed 9 days to equilibrate^35^. Uninoculated bioreactor culture medium used to support microbial communities was also run as a control.

**Figure 1:**
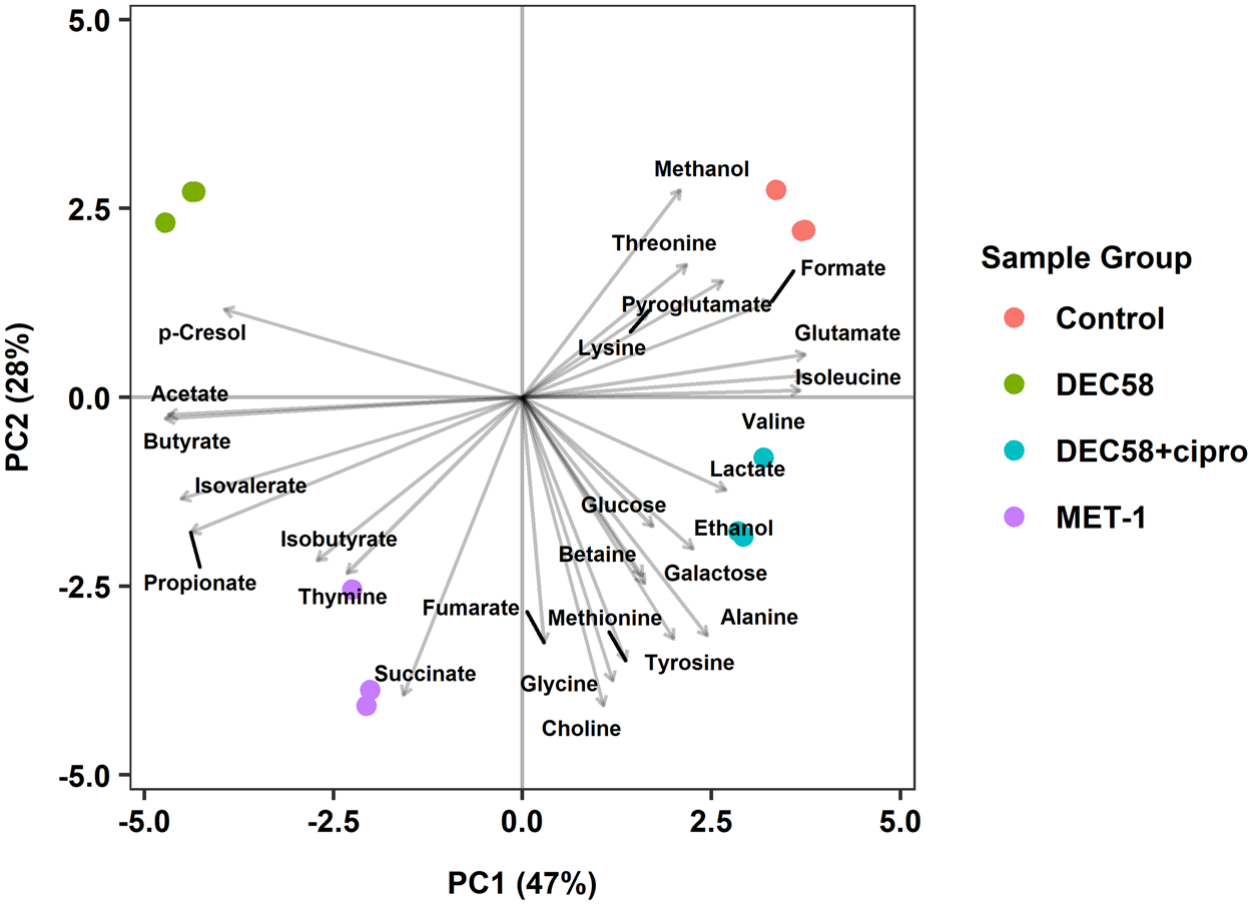
Targeted metabolomics of spent media derived from bioreactor-supported defined microbial ecosystems used in this study. The biplot of the PLS-DA model was generated using mean centered and scaled metabolite concentrations profiled from 1D ^1^H NMR spectroscopy data. Uninoculated bioreactor medium was also profiled as a control. The plot was generated in R using the ropls (version 1.12.0) and ggplot2 (version 2.2.1; H. Wickham. ggplot2: Elegant Graphics for Data Analysis. Springer-Verlag New York, 2016).

Using a targeted approach, metabolite profiles were then generated from the cell-free spent culture supernatant (spent medium) of each microbial ecosystem grown in the bioreactors using 1D ^1^H nuclear magnetic resonance (NMR) spectroscopy. The spent medium derived from individual bioreactor-supported microbial ecosystems all clustered independently by partial least squares discriminate analysis (PLS-DA) (Fig. 1a), indicating that the ecosystems were functionally distinct. The corresponding loadings plot shown in Fig. 1b indicated most metabolites contributed to the explained variation of ecosystem groups within the PLS-DA model.

### *C. difficile* growth is influenced by defined microbial ecosystem-associated components

**Figure 2:**
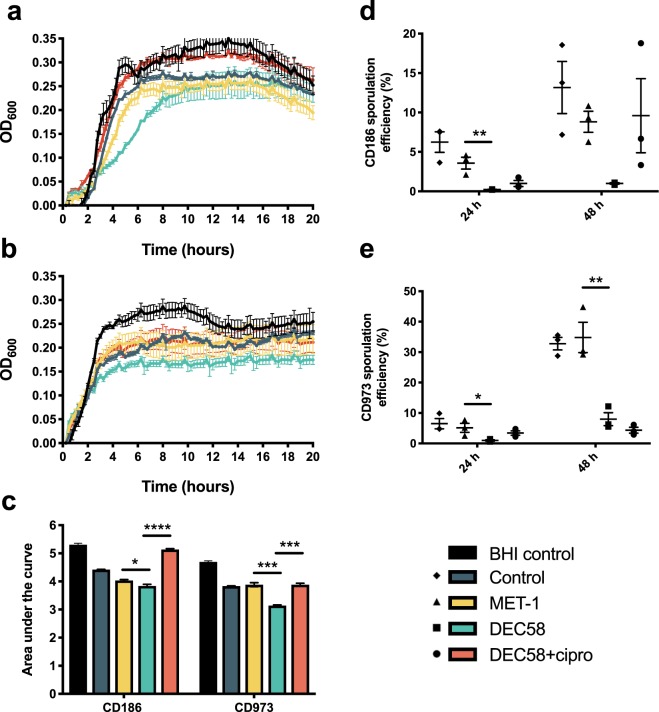
*C. difficile* vegetative cell growth and sporulation efficiency is influenced by defined microbial ecosystem components. CD186 and CD973 were grown BHIS medium containing ethyl acetate-extracted metabolites derived from microbial ecosystems and incubated anaerobically at 37 °C. Vegetative cell growth for CD186 (**a**) and CD973 (**b**) was assessed over 20 h. Area under the curve (AUC) analysis (**c**) was used to quantify the growth of each *C. difficile* strain in response to defined microbial ecosystem components or bioreactor medium components as a control. Sporulation efficiency of CD186 (**d**) and CD973 (**e**) was also determined in response to the spent media from microbial ecosystems. Error bars represent the standard error of the mean from three replicate experiments. *p ≤ 0.05; **p ≤ 0.01; ***p ≤ 0.001; ****p ≤ 0.0001. NB: There was no statistically significant difference in the sporulation efficiency of the CD186 in response to the DEC58 group compared to other treatment groups at 48 h.

To investigate whether *C. difficile* vegetative cell growth could be influenced by microbial ecosystem-extracted metabolite preparations, *C. difficile* vegetative cell growth was measured by OD_600_ over 20 h after exposure to ethyl-acetate extracted metabolites from the spent medium of each defined microbial ecosystem, uninoculated bioreactor control or BHI control (Fig. 2a,b). Using area under the curve (AUC) analysis, outgrowth levels were quantitatively assessed. For both CD186 and CD973 growth, extracted metabolites from DEC58 significantly decreased the mean AUC compared to extracted metabolites from both MET-1 and DEC58 treated with ciprofloxacin (*p* < 0.03) (Fig. 2c).

### *C. difficile* sporulation efficiency is influenced by defined microbial ecosystem spent media

The sporulation efficiency of CD186 and CD973 was quantified after 24 and 48 h co-incubation of defined microbial ecosystem spent media with *C. difficile* cultures. DEC58 spent medium decreased the sporulation efficiency of CD186 and CD973 compared to MET-1 spent medium after 24 h of incubation (*p* < 0.04) (Fig. 2d,e). However, spent medium from ciprofloxacin-treated DEC58 had no significant effect on the sporulation efficiency of CD186 or CD973 compared to untreated DEC58 spent medium after 24 or 48 h (*p* > 0.05).

### Toxin expression by CD186 and CD973 are divergent in their response to microbial ecosystem spent media

**Figure 3:**
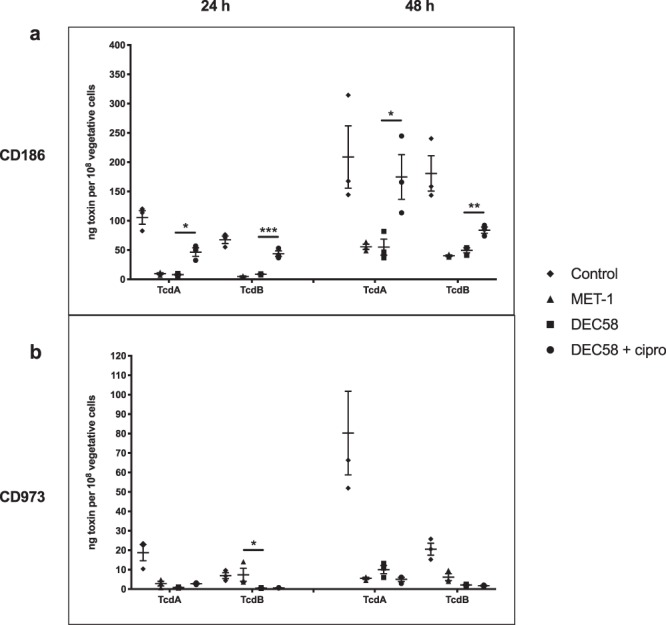
Secreted levels of *C. difficile* TcdA and TcdB are modulated by the spent media of defined microbial ecosystems. TcdA and TcdB levels of CD186 (**a**) and CD973 (**b**) were quantified after 24 and 48 h. Quantities shown are relative to the mean of matched vegetative cell counts for each sample. The mean of three replicate experiments are shown, with error bars showing the standard deviation observed. *p ≤ 0.05; **p ≤ 0.01; ***p ≤ 0.001; ****p ≤ 0.0001.

The levels of secreted TcdA and TcdB were then examined in response to microbial ecosystem spent media. Secreted levels of TcdA and TcdB by CD186 were significantly increased on exposure to ciprofloxacin-treated DEC58 spent medium compared to the untreated DEC58 spent medium (*p* < 0.03) and this effect was also observed after 48 h of treatment (*p* < 0.02) (Fig. 3a). Levels of CD186 TcdA and TcdB did not significantly differ after exposure to either MET-1 or DEC58 spent medium (*p* > 0.05).

Overall, secreted levels of TcdA and TcdB were lower in CD973 compared to CD186 in response to 24 h co-incubation with the medium control (*p* < 0.006). Although CD973 *tcdA* gene expression was increased following exposure to ciprofloxacin-treated DEC58 spent medium compared to untreated DEC58 spent medium after 24 h (see Supplementary Fig. S1), TcdA secretion was not affected by the same conditions (*p* = 0.91). Unlike CD186, TcdA secretion by CD973 did not vary in response to any of the microbial ecosystem spent medium treatments after 24 or 48 h (*p* > 0.05) (Fig. 3b). However, 24 h treatment of CD973 with DEC58 spent medium resulted in lower TcdB secretion compared to that obtained after treatment with MET-1 spent medium (*p* = 0.034).

After 12 h, neither *C. difficile* strain demonstrated changes in *cdtA* or *cdtB* expression following exposure to DEC58 spent medium, even after antibiotic treatment (*p* > 0.05) (see Supplementary Fig. S1). Conversely, after 24 h, ciprofloxacin-treated DEC58 spent medium resulted in an increase of *cdtA* expression compared to DEC58 spent medium in both ribotype strains (*p* < 0.048).

### The metabolic profile of *C. difficile* is modulated in response to defined microbial ecosystem spent media

**Figure 4:**
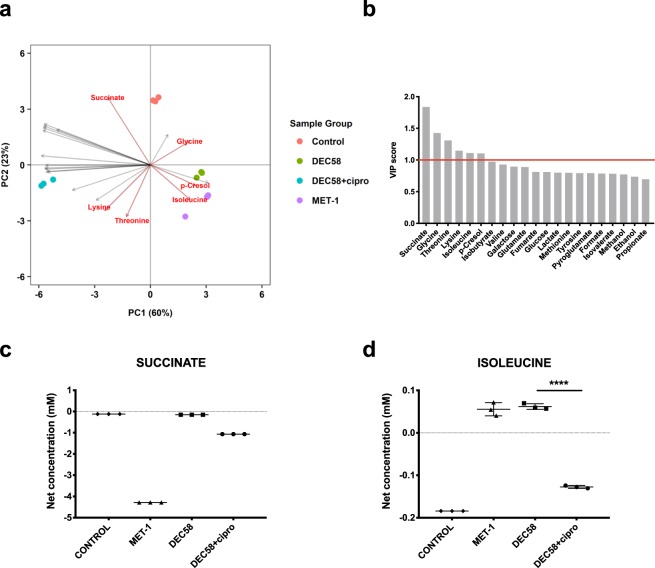
Targeted metabolic response of *C. difficile* CD186 (ribotype 027) after treatment with the spent media of defined microbial ecosystems. The net metabolite output of CD186 was determined by subtracting the mean metabolite concentration data of the microbial ecosystem spent medium from the metabolite data of CD186 treated with each defined microbial ecosystem spent medium after 24 h incubation. A biplot of the supervised clustering using PLS-DA (**a**) was used to visually determine the response of *C. difficile* to each ecosystem grouping. The biplot highlights the significant VIP scores displaying the metabolites that are important to the PLS-DA model (**b**). The net production of succinate (**c**) and isolecuine (**d**) by CD186 in response to defined microbial ecosystem spent media are shown. To determine statistical significance, a one-way ANOVA followed by Tukey’s HSD was used to correct for multiple comparisons when evaluating metabolite concentration data, and FDR adjusted *p-*values are reported. *p ≤ 0.05; **p ≤ 0.01; ***p ≤ 0.001; ****p ≤ 0.0001.

**Figure 5:**
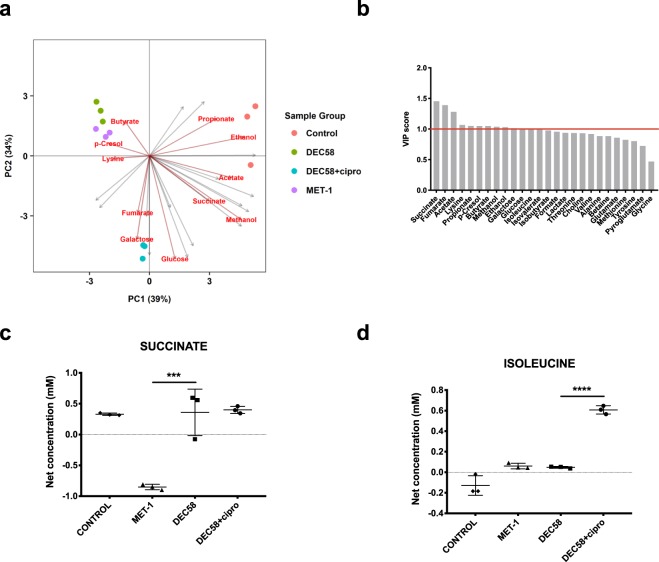
Targeted metabolite response of *C. difficile* CD973 (ribotype 078) after treatment with the spent media of defined microbial ecosystems. The net metabolite output of CD973 was determined by subtracting the mean metabolite concentration data of the microbial ecosystem spent medium from the metabolite data of CD973 treated with each defined microbial ecosystem spent medium after 24 h incubation. A biplot of the supervised clustering using PLS-DA (**a**) was used to visually determine the response of *C. difficile* to each ecosystem grouping. The biplot highlights the significant VIP scores displaying the metabolites that are important to the PLS-DA model (**b**). The net production of succinate (**c**) and isolecuine (**D**) by CD973 in response to defined microbial ecosystem spent media are shown. To determine statistical significance, a one-way ANOVA followed by Tukey’s HSD was used to correct for multiple comparisons when evaluating metabolite concentration data, and FDR adjusted *p-*values are reported. *p ≤ 0.05; **p ≤ 0.01; ***p ≤ 0.001; ****p ≤ 0.0001.

Metabolomic analysis on *C. difficile* co-incubated with spent media from each respective ecosystem (after 24 h) was performed. To investigate if the metabolic response of *C. difficile* was altered upon exposure to the spent media derived from defined microbial ecosystems, we used a subtractive approach to isolate the metabolic influence of *C. difficile* growth from the baseline metabolite contributions provided by each defined microbial ecosystem. For both ribotype strains, treatment with MET-1 and DEC58 spent media resulted in a similar *C. difficile* metabolic response as indicated by close clustering of these groups compared to ciprofloxacin-treated DEC58 and control upon PLS-DA (Figs 4a and 5a). VIP scoring was subsequently conducted on each PLS-DA model to elucidate compounds contributing to the total variation of *C. difficile* metabolic response (Figs 4b and 5b). Compounds significant by VIP scoring (VIP ≥ 1) for both ribotype strains included: succinate, lysine, and *p-*cresol. CD186-specific compounds that were significant by VIP scoring included: succinate, glycine, threonine, lysine, isoleucine and *p*-cresol (Fig. 4b). CD973-specific compounds that were significant by VIP scoring included: succinate, fumarate, acetate, lysine, propionate, *p*-cresol, butyrate, methanol, ethanol, galactose and glucose (Fig. 5b). The net metabolic output of succinate and isoleucine by CD186 and CD973 are shown in Figs 4c,d and 5c,d, respectively. Net metabolite concentrations for the remaining compounds profiled are shown in Supplementary Fig. S2.

## Discussion

Given the importance of the normal gut microbiota in the context of rCDI, this study aimed to examine the influence of microbiota-derived components on *C. difficile* growth and virulence determinants. To do this, *C. difficile* was challenged with components from the MET-1, DEC58 and ciprofloxacin-treated DEC58 defined microbial ecosystems supported in bioreactors, and the biological response of two distinct, epidemic-associated ribotype strains of *C. difficile* were subsequently examined.

As extracted metabolites derived from the ciprofloxacin-treated DEC58 ecosystem promoted increased vegetative cell growth in both ribotype strains of *C. difficile* compared to untreated DEC58, we hypothesized that metabolites associated with this ecosystem may be associated with the modulation of *C. difficile* virulence determinants. Subtractive metabolomic analysis allowed for the determination of the net metabolomic output of *C. difficile* in response to components from the defined microbial ecosystems.

VIP scoring elucidated that succinate significantly contributed to the total variation of *C. difficile* metabolic response. Interestingly, succinate was produced by all three ecosystems, but was not detected in co-incubation with CD186, indicating that CD186 readily consumed succinate in response to components from MET-1, DEC58, and ciprofloxacin-treated DEC58 spent media treatments. Also, the initial levels of succinate in DEC58 spent medium (mean 0.153 mM) was lower than the ciprofloxacin-treated DEC58 spent medium (mean 1.067 mM), where there was also an association of increased monosaccharides and branched-chain amino acids with the ciprofloxacin-treated condition compared to the untreated DEC58 community (Fig. 1). This suggested a shift in metabolism from carbohydrate to proteolytic fermentation. The depletion of succinate-utilizing microbes (secondary fermenters of sugars/sugar alcohols) such as species within the Clostridiaceae family, e.g. *Clostridium saccharobutylicum*, may have resulted in the increased levels of free succinate^36^.

A recent study has shown that upon antibiotic perturbation in mice, levels of succinate produced by the gut microbiota are increased and become available for *C. difficile* to use as a growth substrate to efficiently expand in the intestine^5^. Therefore, the increased levels of free succinate in ciprofloxacin-treated DEC58 spent medium may have enabled consumption by CD186. On the other hand, in the untreated DEC58 community, cross-feeding mechanisms between microbial species likely keep succinate levels low, precluding *C. difficile* from metabolizing excess amounts of this compound. Similarly, the spent medium of MET-1 contained increased levels of succinate compared to DEC58, which was fully depleted upon incubation with both CD186 and CD973. *C. difficile* vegetative cell growth experiments further corroborated the findings on succinate utilization as exposure to ciprofloxacin-treated DEC58 metabolites resulted in increased CD186 growth compared to exposure to DEC58 metabolites alone. Similarly, the high levels of succinate found in MET-1 spent media may have contributed to the increased vegetative cell growth of both *C. difficile* strains in response to treatment with metabolites of MET-1 compared to DEC58.

Previous work conducted in collaboration with our laboratory found that a live MET-1 bacterial preparation was protective against *C. difficile* TcdA-mediated cytotoxicity in an antibiotic-induced colitis murine model^33^. Given the importance of *C. difficile* toxins to the pathogenesis of CDI, we hypothesized that MET-1 and DEC58 components may mediate therapeutic effects through antagonistic mechanisms on the expression, regulation, or production of *C. difficile* TcdA and TcdB independent of host-associated factors. An important finding from this study was that CD186 (ribotype 027) TcdA and TcdB levels were significantly increased upon treatment with the spent medium derived from ciprofloxacin-treated DEC58 compared to untreated DEC58. This suggested that a perturbed microbial ecosystem may be impaired in its ability to antagonize *C. difficile* toxin production compared to its unperturbed counterpart. While succinate consumption by CD186 was shown to occur in response to the spent media from all three ecosystems examined, the relationship of this compound to toxin expression is currently unclear, as only ciprofloxacin-treated DEC58 spent medium was shown to significantly augment toxin production in this ribotype strain.

Current evidence suggests that toxin production by *C. difficile* is a highly complex process orchestrated in response to several environment cues related to the availability of growth substrates^37,38^. Thus, it is likely that *C. difficile* toxin expression is regulated by the synergistic activity of several metabolites produced by defined microbial ecosystems and/or that these effects may be strain-dependent. Interestingly, the amino acid isoleucine was efficiently consumed by CD186 but not CD973 upon treatment with spent medium from ciprofloxacin-treated DEC58 compared to spent medium from untreated DEC58. As specific amino acid supplementation with isoleucine has been shown to increase toxin expression in cultures of *C. difficile* VPI 10463 (ribotype 087)^39^, the consumption of isoleucine by CD186 may explain the increased toxin expression observed. Additionally, limited levels of glucose in defined culture media has also been shown to result in reduced *C. difficile* toxin yield^39^. High levels of glucose in the spent medium of ciprofloxacin-treated DEC58 compared to DEC58 may have similarly contributed to the augmented toxin production observed.

As well as TcdA and TcdB, we also sought to understand the effect of gut microbial ecosystems on an additional *C. difficile* toxin, CDT, which has been associated with highly virulent *C. difficile* isolates^40–42^. While we did not investigate CDT secretion, we found that spent medium from DEC58 was able to repress the expression of CD186 and CD973 *cdtA* compared to spent medium from DEC58 after 24 h (see Supplementary Fig. S1). The ability of ciprofloxacin-treated DEC58 to increase *cdtA* expression may be linked with the ability of CDT-expressing *C. difficile* to downregulate the host immune response and augment its virulence in the antibiotic-perturbed gut environment^40^.

When comparing the effects of extracted metabolites of either DEC58 or its subset community, MET-1, on *C. difficile* sporulation, we found that the extract from DEC58 was able to decrease the sporulation efficiency of CD186 and CD973 compared to MET-1. Clinically, the ability for a microbial ecosystem to decrease *C. difficile* sporulation efficiency would be favourable to limit pathogen transmissibility and effectively manage disease recurrence^43^. While the environmental signals necessary to initiate sporulation in *C. difficile* are not fully understood^44^, future studies examining the secreted compounds associated with DEC58 spent medium may elucidate triggers of this complex process.

In this study, we used two *C. difficile* isolates representative of two distinct ribotypes that have been implicated in hospital and community-acquired infections^45–47^. Both CD186 and CD973 displayed heterogeneous results in respect to metabolic output and virulence determinants in response to individual defined microbial ecosystem components tested. For example, while we found that the levels of TcdA or TcdB secreted by CD186 (ribotype 027) were increased by treatment with the spent medium derived from ciprofloxacin-treated DEC58 compared to untreated DEC58, toxin levels were not influenced in CD973 (ribotype 078) under the same conditions. Several recent studies in mouse models have shown that an individual isolate of *C. difficile* is capable of modulating its physiology to adapt to different nutrient niches within diverse gut microbiome landscapes^48–50^. Our results support these findings and further suggest that there is stain-to-strain variability in substrate utilization by *C. difficile*. Although ribotype is not predictive of increased disease severity or clinical outcome^51,52^, future studies investigating the contrasting manner by which distinct *C. difficile* ribotypes are able to capitalize on the antibiotic-perturbed gut environment will provide valuable insight into ecological mechanisms involved in the development and persistence of CDI. Additionally, as the incidence of BI/NAP1/027 isolates has steadily decreased in the United Kingdom and other areas of Western Europe since the late 2000s^53^, the varied distribution of *C. difficile* strains worldwide will need to be considered for the development of novel microbiota-based therapeutics.

The results from this study suggest that defined microbial ecosystem components are able to modulate *C. difficile* growth and virulence determinants *in vitro*. Future studies examining the precise components of microbial ecosystems responsible for these antagonistic properties will help advance the development of tailored microbiome therapeutics for the effective treatment of rCDI.

## Materials and Methods

### Development of defined microbial ecosystems

A detailed explanation of the protocol for culturing microorganisms from donor stool has been previously described^32^. Briefly, stool from a healthy 41-year old female donor was diluted in sterile pre-reduced saline and plated onto a selection of agar plates each optimal for a different group of bacterial species. The selected donor had a healthy lifestyle, normal body mass index (BMI), and experienced very few antibiotic exposures in childhood and within the ten years previous to donation. Strains isolated from donor stool were purified by repeated plate subculture under strict anaerobic conditions at 37 °C and subsequently stored at −80 °C. Genomic DNA was extracted from all isolates using the Maxwell® 16 DNA purification kit (Promega, Madison, Wisconsin, USA) and identified by sequencing of the 16S rRNA gene using V3kl and V6r primers (Supplementary Table S2) under the following cycling conditions: 94 °C for 2 min, 94 °C for 30 s, 60 °C for 30 s, and 72 °C for 30 s repeated for 30 cycles, followed by a final elongation at 72 °C for 5 min. Species identifications were determined using NCBI BLAST (https://www.ncbi.nlm.nih.gov/BLAST/).

Purified strains were individually cultured on fastidious anaerobe agar (Lab M Ltd. Heywood, Lancashire, UK) with or without 5% defibrinated sheep’s blood (Hemostat Laboratories, Dixon, California) under anaerobic conditions at 37 °C for 48 h. Bacterial isolates specific to each respective community were resuspended in 50 mL of pre-reduced 0.9% saline before inoculation into a single-stage bioreactor vessel (Infors, Switzerland).

The development and operation of bioreactor vessels to model communities derived from human fecal ecosystems, including the bioreactor culture medium composition, have been described elsewhere^54^. Briefly, a 400 mL bioreactor vessel was cultured under flow (chemostat) conditions to support the growth of each microbial ecosystem. Culture vessels were maintained under anaerobic conditions through the continuous sparging of N_2_ gas, and held at 37 °C and a pH of 6.9–7.0 to mimic the conditions of the human distal gut. The culture medium was added at a rate of 400 mL/day to give a retention time of 24 h, and contents were gently stirred to ensure even nutrient dispersion. Vessel contents were sampled daily and harvested 9 days post-inoculation. Immediately following the bioreactor run, vessel contents were ultracentrifuged at 92,387 × *g* for 2 h at 4 °C and the clarified supernatants were filtered through a 0.22 µm sterile filter. The resulting, cell-free spent medium from each defined microbial ecosystem was stored at −20 °C until use in further experiments.

MET-1 and DEC58 (Supplementary Table S1) communities were constructed from a subset of strains cultured from donor stool. The ciprofloxacin-treated DEC58 community was created by culturing the ecosystem in bioreactors and pulse-dosing with 137.5 mg of ciprofloxacin (Sigma, dissolved in a 0.1 N HCl solution, 5.5 mL of 25 mg/mL) every 12 h starting at day 7 post-inoculation. The final concentration of ciprofloxacin within the bioreactor after dosing was 0.344 mg/mL. The quantity of ciprofloxacin used to dose the chemostat vessels was derived from the percent fecal-excretion (27.5%) of a 500 mg oral dose of ciprofloxacin in human subjects^55^. Given that there is no absorption of antibiotic within the chemostat vessel, the measure of fecal-excretion is suitable to estimate the physiologically relevant doses of ciprofloxacin in this model.

### *C. difficile* growth curves in response to defined microbial ecosystem components

Bioreactor vessel contents were collected on day 9, and immediately frozen at −20 °C. Small molecules were extracted from these samples by adding an equal volume of ethyl acetate (Sigma), shaking vigorously and allowing to settle and then repeating the procedure. The aqueous organic solvent phase was removed using glass-pipettes and transferred to separate glass bottles. Silicone tubing connected to an air-line was used to accelerate the rate of evaporation of the solvent phase, and the remaining dried extracts were stored at −20 °C. Dried extracts were resuspended in equal volumes of brain-heart infusion (BHI) broth, filter-sterilized through a 0.22 µm filter using a vacuum and stored at 4 °C. Purified spore stocks of CD186 and CD973 (see Supplementary Table S3) were allowed to germinate in BHI supplemented with 2 mM of sodium taurocholate (Sigma). *C. difficile* vegetative cells were then diluted 1:5 in defined microbial ecosystem liquid extracts in a 96-well plate and incubated anaerobically at 37 °C in a Victor^3^ plate reader (Perkin Elmer, USA). Growth measurements (OD_600_) were automatically recorded over 20 h. The area under the curve (AUC) was calculated from average OD_600_ data (biological triplicate) from *x* = 0–20 using a baseline of *y* = 0. Statistical significance was determined in comparison to the DEC58 group (used as a control) using a one-way ANOVA followed by Dunnett’s multiple comparisons test.

### *C. difficile* vegetative cell counts

*C. difficile* ribotype 027 (CD186) and 078 (CD973) strains were spread onto brain-heart infusion (BHI) agar supplemented with 0.1% sodium taurocholate (Sigma), yeast-extract, and _L_-cysteine (BHIS agar) from −80 °C freezer stocks and incubated anaerobically at 37 °C for 72 h. Cell-free spent medium from defined microbial ecosystems were inoculated in an equal volume with CD186 or CD973 culture grown to OD_600_ = 0.1–0.2 in BHI broth. Samples were incubated anaerobically at 37 °C for 24 or 48 h.

### Determination of *C. difficile* sporulation efficiency

Total *C. difficile* cell counts (cells/mL) were generated by serially diluting and plating 0.1 mL of each treated culture on BHIS agar. Spore counts were generated by treating 0.5 mL of spent medium-treated *C. difficile* cultures with an equal volume of 100% ethanol for 1 h at room temperature. Samples were then pelleted by centrifugation at 14,000 × *g* for 5 min, and the supernatant was removed. Spore-containing pellets were then resuspended in 0.5 mL of phosphate-buffered saline (PBS), serially diluted and plated on BHIS agar. Plates were incubated anaerobically at 37 °C and enumerated after 24 and 48 h. Sporulation efficiency was calculated as a percent of the spore cell count divided by the total cell count for each sample. Mean bacterial cell counts were determined from three technical replicate spread-plating experiments and statistical significance was determined in comparison to the DEC58 group (used as a control) using a one-way ANOVA followed by Dunnett’s multiple comparisons test.

### *C. difficile* TcdA and TcdB secretion assay

Approximately 10 mL of spent medium treated *C. difficile* cultures were spun down at 4,686 × *g* for 10 min and the resulting supernatants were sterile-filtered. Separate quantification of TcdA and TcdB *C. difficile* toxins was completed using the TGC E002-1 toxin ELISA kit (tgcBIOMICS, Bingen, Germany) following manufacturer’s instructions. Two-fold serial dilutions of TcdA and TcdB standard recombinant toxins were included on each ELISA plate to generate a toxin calibration curve. Toxin quantification levels were normalized to matched vegetative cell count data at 24 and 48 h for each biological replicate. Normality was assessed using the D’Agostino & Pearson normality test. To determine statistical significance relative to the DEC58 group (used as a control), a one-way ANOVA followed by Dunnett’s multiple comparisons test was performed on normally distributed data, and a Kruskal-Wallis test followed by Dunn’s multiple comparison test was used for non-normally distributed data.

### 1D ^1^H NMR spectroscopy

Sterile-filtered cell-free spent media from defined microbial ecosystems were prepared for 1D ^1^H NMR scanning as previously described^35^. Briefly, samples were diluted to 10% (v/v) and combined with the internal standard. The internal standard consisted of 5 mM 4,4-dimethyl-4-silapentane-1-sulfonic acid (DSS) and 0.2% (w/v) sodium azide preservative, dissolved in 99.9% D_2_O. Samples were then transferred to Wilmad 5 mm glass NMR tubes (Sigma), and stored at 4 °C overnight. Samples were allowed to return to room temperature before scanning on a Bruker Avance 600 MHz spectrometer, housed at the NMR Centre (Advanced Analysis Center, University of Guelph). All NMR spectra were obtained using the first increment of a 1D NOESY pulse sequence with a mixing time of 100 ms, 3.89 s acquisition time, and 3 s relaxation delay for adequate water suppression. Spectra were acquired with 32 scans, and each experiment ran for approximately 4 min. Sample pH was measured immediately after scanning using colourimetric pH indicator strips (Whatman).

Spectra files were processed in their entirety using Chenomx NMR Suite 8.5 (Chenomx Inc., Edmonton, Canada). Briefly, all spectra were phase and baseline corrected using the Chenomx automatic tools with subsequent manually adjustment when required. Targeted profiling was used to identify and quantify compounds in the pre-processed NMR spectra. Briefly, a library of 27 compounds was generated from the internal Chenomx/Bruker’s 600 MHz compound library. Compounds were profiled based on a set of criteria previously described^35^. Briefly, compounds were fit to spectra based on their signatures and properties obtained from the Chenomx software database, as calibrated to the internal standard, DSS. Metabolite concentrations were generated from the area of the projected signal after it was fit to the peak centers during identification.

The PLS-DA model including loadings plots were generated from mean centered and unit variance scaled concentration data. VIP scores were generated from raw concentration data. PLS-DA and loadings plots were generated in R using the ropls (version 1.12.0)^56^ and ggplot2 (version 2.2.1)^57^ packages. To determine statistical significance, a one-way ANOVA followed by Tukey’s HSD was used to correct for multiple comparisons when evaluating metabolite concentration data, and false discovery rate (FDR) adjusted *p-*values were calculated using MetaboAnalyst^58^.

All graphs and statistical analyses were performed using Graph Pad Prism 7.0 (GraphPad Software, La Jolla California USA, www.graphpad.com), unless otherwise stated.

### Ethics Statement

Written informed consent was obtained from the stool donor. This study was approved by the Research Ethics Board at the University of Guelph (#10JL002) and meets the principles of the Helsinki Declaration of the World Medical Association. All donors provided informed consent.

## Supplementary information

Supplementary Information

## Data Availability

Data files from this study are openly available from the Figshare online digital repository. Accession links: https://figshare.com/s/12bb50c7c2ed276c1356, https://figshare.com/s/134e56586183ee774000.
