# Supplementary material for: Effects of defined gut microbial ecosystem components on virulence determinants of Clostridioides difficile
Source: Sci Rep. 2019 Jan 29;9:885. doi: 10.1038/s41598-018-37547-x (PMC6351598; doi:10.1038/s41598-018-37547-x)
Supplement: Supplementary file 1 — Supplementary Information [file 41598_2018_37547_MOESM1_ESM.pdf]

# Supplementary Information:

**Effects of defined gut microbial ecosystem components on virulence determinants of *Clostridioides difficile***

Christian Carlucci[1*], Carys S. Jones[1], Kaitlyn Oliphant[1], Sandi Yen[1], Michelle Daigneault[1], Charley Carriero[1], Avery Robinson[1], Elaine O. Petrof[2], J Scott Weese[3], Emma Allen-Vercoe[1]

[1] Department of Molecular and Cellular Biology, University of Guelph, Guelph, Ontario, Canada

[2] Division of Infectious Diseases/Gastrointestinal Diseases Research Unit, Department of Medicine, Queen's University, Kingston, Ontario, Canada

[3] Department of Pathobiology, Ontario Veterinary College, University of Guelph, Guelph, Ontario, Canada

*Corresponding author: Christian Carlucci

Email: ccarlucc@uoguelph.ca

**Supplementary Table 1. List of cultured bacterial isolates used to generate the MET-1 and DEC58 communities used in this study.** All species listed were included in the DEC58 ecosystem, while species highlighted in bold were included in MET-1. Closest species match was inferred by alignment of 16S rRNA gene sequences to NCBI BLAST.

| | Species identification: | Note: |
|---|---|---|
| 1 | ***Acidaminococcus intestini*** | |
| 2 | *Akkermansia muciniphila* | |
| 3 | *Alistipes finegoldii* | |
| 4 | *Atopobium minutum* | |
| 5 | *Bacteroides caccae* | |
| 6 | *Bacteroides dorei* | |
| 7 | *Bacteroides eggerthii* | |
| 8 | *Bacteroides fragilis* | |
| 9 | ***Bacteroides ovatus*** | |
| 10 | *Bacteroides timonensis/cellulosilyticus* | |
| 11 | *Bacteroides uniformis* | |
| 12 | *Bacteroides vulgatus* | |
| 13 | ***Bifidobacterium adolescentis/faecale/stercoris*** | 2 different strains included in MET-1 |
| 14 | ***Bifidobacterium longum/breve*** | 3 different strains included in MET-1 |
| 15 | ***Blautia luti*** | 2 different strains included in MET-1 |
| 16 | *Blautia producta/coccoides* | |
| 17 | ***Blautia stercoris*** | |
| 18 | ***Butyricicoccus faecihominis/Agathobaculum butyriciproducens*** | |
| 19 | *[Clostridium] aldenense* | |
| 20 | *[Clostridium] citroniae* | |
| 21 | *[Clostridium] lactatifermentans* | |
| 22 | *[Clostridium] oroticum* | |
| 23 | ***Clostridium saccharobutylicum*** | |
| 24 | *[Clostridium] saccharogumia* | |
| 25 | *[Clostridium] scindens* | |
| 26 | ***Collinsella aerofaciens*** | |
| 27 | *Coprococcus catus* | |
| 28 | *Coprococcus comes* | |
| 29 | *Dielma fastidiosa* | |
| 30 | *Dorea formicigenerans* | |
| 31 | ***Dorea longicatena*** | 2 different strains included in MET-1 |
| 32 | *Eggerthella lenta* | |
| 33 | ***Escherichia coli*** | |
| 34 | *[Eubacterium] contortum* | |
| 35 | ***[Eubacterium] eligens*** | |
| 36 | ***Eubacterium limosum/aggregans/callanderi*** | |
| 37 | ***[Eubacterium] rectale*** | 3 different strains included in MET-1 |
| 38 | ***[Eubacterium] ventriosum*** | |
| 39 | ***Faecalibacterium prausnitzii*** | |
| 40 | *Flavonifractor plautii* | |
| 41 | *Hungatella effluvii* | |
| 42 | ***Klebsiella aerogenes*** | |
| 43 | ***Lachnospira pectinoschiza*** | |
| 44 | ***Lactobacillus casei*** | |
| 45 | ***Lactobacillus paracasei*** | |
| 46 | *Lactonifactor longoviformis* | |
| 47 | *Neglecta timonensis* | |
| 48 | *Oscillibacter ruminantium* | |
| 49 | ***Parabacteroides distasonis*** | |
| 50 | *Parabacteroides merdae* | |
| 51 | *Phascolarctobacterium faecium* | |
| 52 | ***Roseburia faecis*** | |
| 53 | ***Roseburia intestinalis*** | |
| 54 | ***[Ruminococcus] faecis*** | 2 different strains included in MET-1 |
| 55 | *[Ruminococcus] gnavus* | |
| 56 | ***Streptococcus rubneri/parasanguinis/australis*** | |
| 57 | *Veillonella dispar* | |
| 58 | *Veillonella parvula/tobetsuensis/rogosae* | |

## Supplementary Table 2. Properties of *C. difficile* isolates used in this study

| Strain: | Ribotype: | Toxinotype: | Toxins encoded: | PFGE* type: | Source**: |
|---|---|---|---|---|---|
| CD186 | 027 | III | A+B+CDT+ | NAP 1 | Sherbrooke, Quebec, 2003 (outbreak) |
| CD973 | 078 | V | A+B+CDT+ | NAP 7 | Brantford, Ontario, 2008 (non-outbreak) |

* PFGE = pulsed-field gel electrophoresis
** All isolates were obtained from J. Scott Weese at the University of Guelph, Ontario, Canada.

**Supplementary Table 3. Primers used in this study.** Primers used to sequence the variable regions of the 16S rRNA gene and primers used in RT-qPCR to assess the effects of defined microbial ecosystems on *C. difficile* toxin gene expression.

| Name: | Amplicon size (bp): | Primer concentrations (nM): | Primer Sequence (5'-3'): | Reference: |
|---|---|---|---|---|
| V3kl/V6r 16S rRNA | 735 | 500 | F'-TACGG[AG]AGGCAGCAG<br>R'-AC[AG]ACACGAGCTGACGAC | Gloor et al., 2010 |
| *tcdA* (Toxin A) | 56 | 500 | F'-TGTCAGAAGCTCGCTCCACA<br>R'-AGCTGACGCATAAGCTCCTGGAC | This study |
| *tcdB* (Toxin B) | 167 | 500 | F'-CCTGGAGATGGTGAAATAC<br>R'-GCTGCTTCTATTTCTGTGG | Metcalf, 2012 |
| *cdtA* (Binary toxin enzymatic) | 81 | 500 | F'-TGCAATACTACTTACAAGGCTCCTATAGA<br>R'-TCTTTCCCATTCTTTAGCCTTTTC | Carter et al., 2007 |
| *rrs* (16S ribosmoal RNA) | 120 | 500 | F'-GGGAGACTTGAGTGCAGGAG<br>R'-GTGCCTCAGCGTCAGTTACAGT | Denève et al., 2008 |

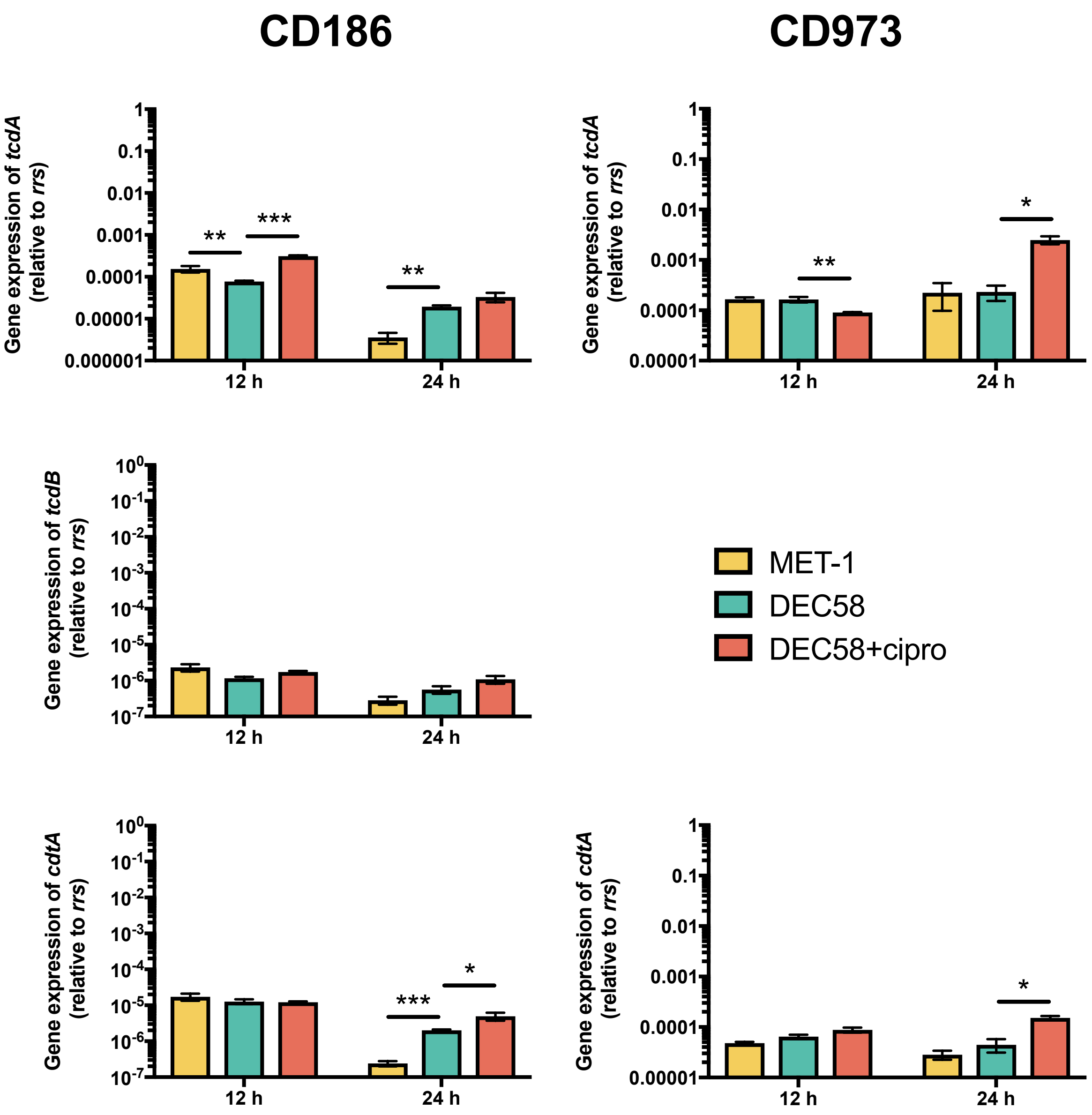


**Supplementary Figure 1.** ***C. difficile*** **toxin gene expression in response to the spent-media of defined microbial ecosystems.** Of note, CD973 *tcdB* expression could not be determined as the $C_q$ values were below the detection limit of qPCR. Error bars represent the standard deviation of three biological replicate experiments run in technical triplicate. *, $p \leq 0.05$; **, $p \leq 0.01$; ***, $p \leq 0.001$; ****, $p \leq 0.0001$.

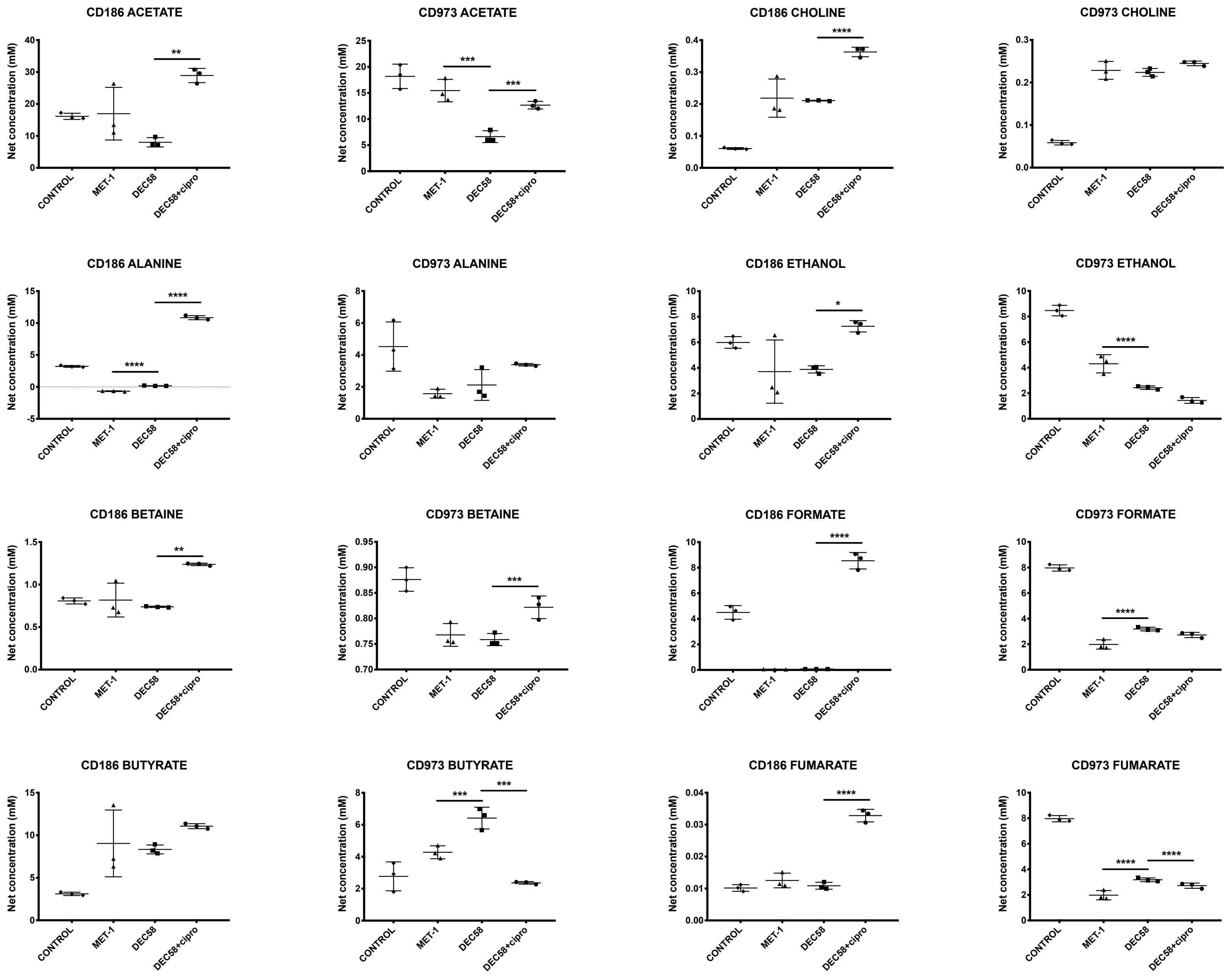

CD186 ACETATE
Net concentration (mM)
0
10
20
30
40
**
CONTROL
MET-1
DEC58
DEC58+cipro
CD973 ACETATE
Net concentration (mM)
0
5
10
15
20
25
***
***
CONTROL
MET-1
DEC58
DEC58+cipro
CD186 CHOLINE
Net concentration (mM)
0.0
0.1
0.2
0.3
0.4
****
CONTROL
MET-1
DEC58
DEC58+cipro
CD973 CHOLINE
Net concentration (mM)
0.0
0.1
0.2
0.3
CONTROL
MET-1
DEC58
DEC58+cipro
CD186 ALANINE
Net concentration (mM)
-5
0
5
10
15
****
****
CONTROL
MET-1
DEC58
DEC58+cipro
CD973 ALANINE
Net concentration (mM)
0
2
4
6
8
CONTROL
MET-1
DEC58
DEC58+cipro
CD186 ETHANOL
Net concentration (mM)
0
2
4
6
8
10
*
CONTROL
MET-1
DEC58
DEC58+cipro
CD973 ETHANOL
Net concentration (mM)
0
2
4
6
8
10
****
CONTROL
MET-1
DEC58
DEC58+cipro
CD186 BETAINE
Net concentration (mM)
0.0
0.5
1.0
1.5
**
CONTROL
MET-1
DEC58
DEC58+cipro
CD973 BETAINE
Net concentration (mM)
0.70
0.75
0.80
0.85
0.90
0.95
***
CONTROL
MET-1
DEC58
DEC58+cipro
CD186 FORMATE
Net concentration (mM)
0
2
4
6
8
10
****
CONTROL
MET-1
DEC58
DEC58+cipro
CD973 FORMATE
Net concentration (mM)
0
2
4
6
8
10
****
CONTROL
MET-1
DEC58
DEC58+cipro
CD186 BUTYRATE
Net concentration (mM)
0
5
10
15
CONTROL
MET-1
DEC58
DEC58+cipro
CD973 BUTYRATE
Net concentration (mM)
0
2
4
6
8
***
***
CONTROL
MET-1
DEC58
DEC58+cipro
CD186 FUMARATE
Net concentration (mM)
0.00
0.01
0.02
0.03
0.04
****
CONTROL
MET-1
DEC58
DEC58+cipro
CD973 FUMARATE
Net concentration (mM)
0
2
4
6
8
10
****
****
CONTROL
MET-1
DEC58
DEC58+cipro

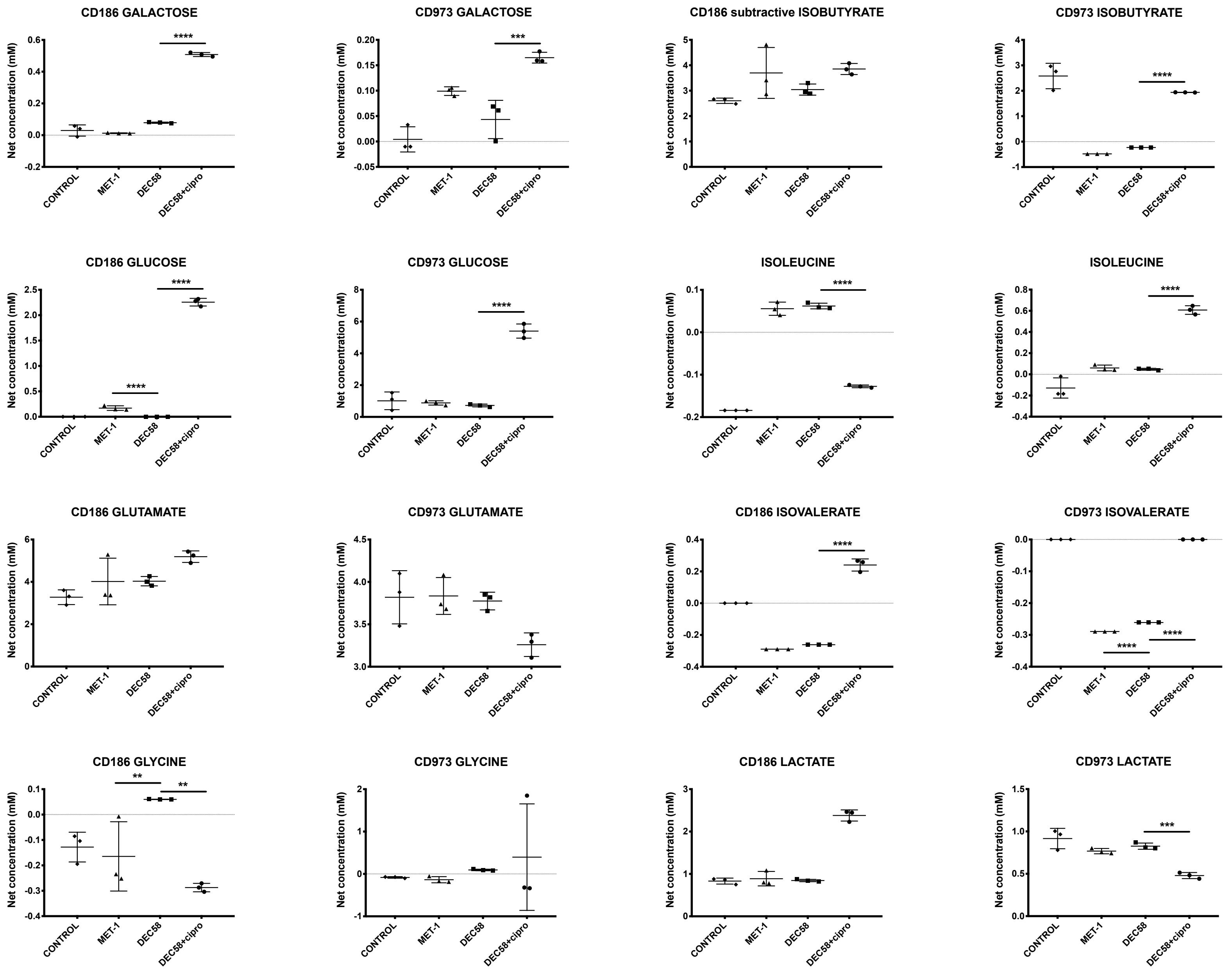
CD186 GALACTOSE
CD973 GALACTOSE
CD186 subtractive ISOBUTYRATE
CD973 ISOBUTYRATE
CD186 GLUCOSE
CD973 GLUCOSE
ISOLEUCINE
ISOLEUCINE
CD186 GLUTAMATE
CD973 GLUTAMATE
CD186 ISOVALERATE
CD973 ISOVALERATE
CD186 GLYCINE
CD973 GLYCINE
CD186 LACTATE
CD973 LACTATE
Net concentration (mM)
CONTROL
MET-1
DEC58
DEC58+cipro

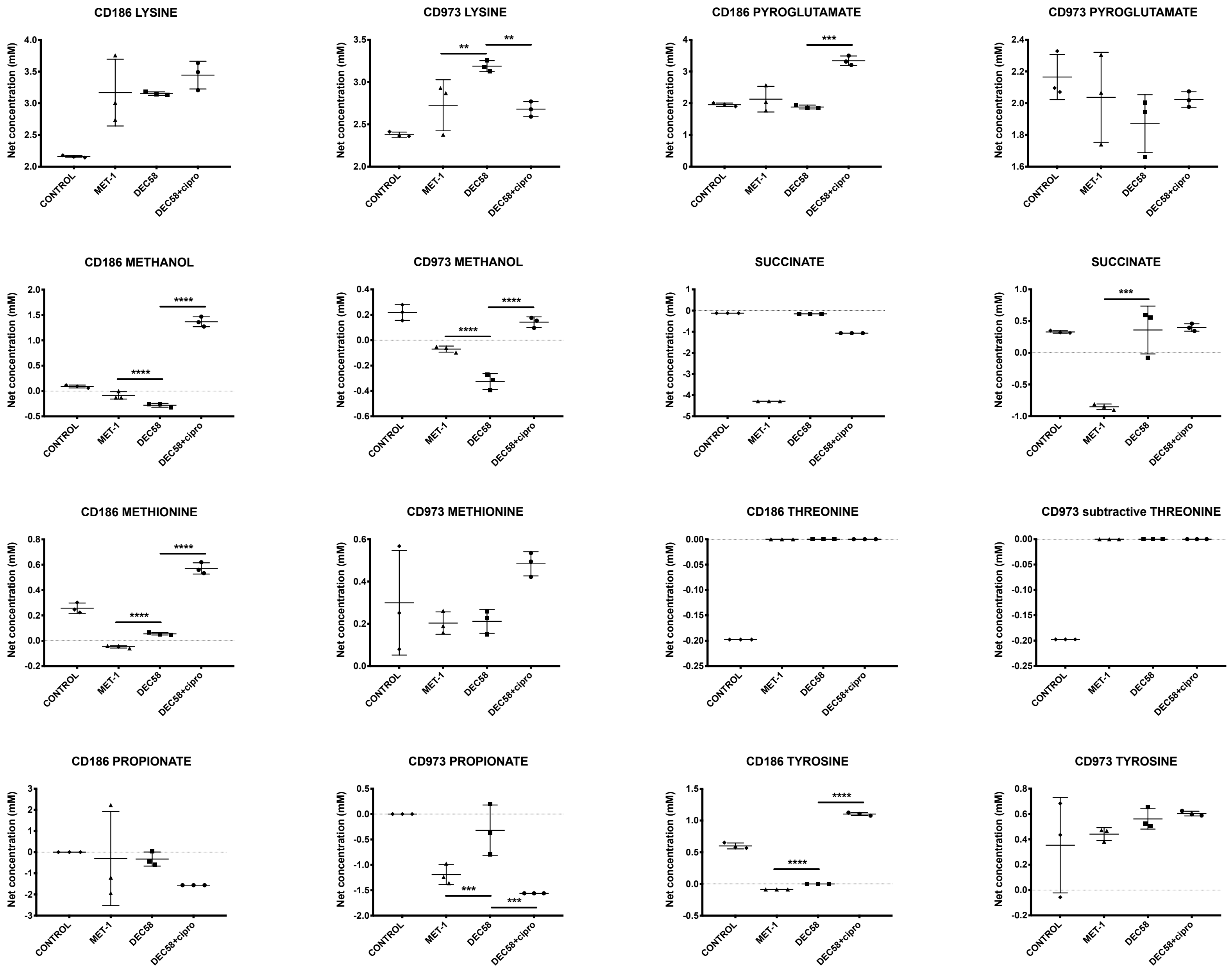
CD186 LYSINE
CD973 LYSINE
CD186 PYROGLUTAMATE
CD973 PYROGLUTAMATE
CD186 METHANOL
CD973 METHANOL
SUCCINATE
SUCCINATE
CD186 METHIONINE
CD973 METHIONINE
CD186 THREONINE
CD973 subtractive THREONINE
CD186 PROPIONATE
CD973 PROPIONATE
CD186 TYROSINE
CD973 TYROSINE
Net concentration (mM)
CONTROL
MET-1
DEC58
DEC58+cipro

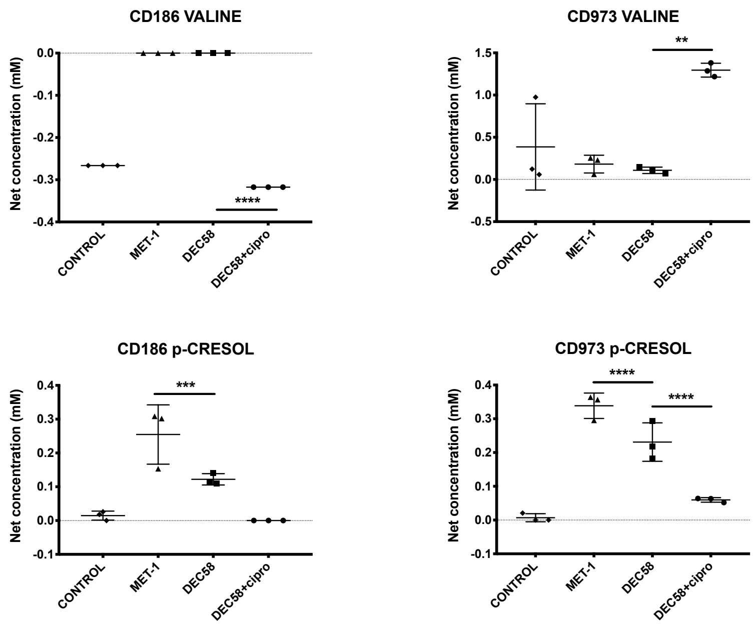


**Supplementary Figure 2. Targeted metabolite response of *C. difficile* CD186 and CD973 after treatment with the spent-media of defined microbial ecosystems.** The net metabolomic output of CD186 and CD973 was respectively determined by subtracting the mean metabolite concentration data of the bioreactor-supported ecosystem spent-medium from the metabolite data of *C. difficile* treated with each defined microbial ecosystem spent-medium after 24 h incubation. All compounds were determined using 1D $^1$H NMR spectroscopy. To determine statistical significance, a one-way ANOVA followed by Tukey's HSD was used to correct for multiple comparisons when evaluating metabolite concentration data, and FDR adjusted *p*-values are reported. Only statistical comparisons between MET-1 and DEC58; DEC58 and DEC58+cipro groups are shown. Error bars represent the standard error of the means for three replicate experiments. *, $p \leq 0.05$; **, $p \leq 0.01$; ***, $p \leq 0.001$; ****, $p \leq 0.0001$.

## Supplementary Materials and Methods:

***C. difficile* toxin gene expression assay.** Cell-free spent medium from defined microbial ecosystems were inoculated in an equal volume with CD186 or CD973 culture grown to $OD_{600}$ = 0.1-0.2 in BHI broth. Samples were incubated anaerobically at 37ºC for 12, 24 or 48 h. Approximately 10 mL of spent-medium treated *C. difficile* cultures were pelleted by centrifugation at 4,686 × *g* for 10 min at 4ºC. RNA-containing pellets were stabilized using 1 mL of RNAprotect reagent (Qiagen) according to manufacturer's instructions and were frozen at -80ºC. Total bacterial RNA was isolated and purified using the RNeasy Mini kit (Qiagen, Mississauga, Ontario, Canada) following manufacturer's instructions with some modifications. Briefly, RNAprotect-treated pellets were resuspended in 1 mL of RLT lysis buffer (Qiagen) + 0.01% (v/v) β-mercaptoethanol and lysed with 200 ± 10 mg of 0.1 mm zirconium beads using a bead-beater homogenizer (Digital Disrupter Genie, Scientific Industries Inc., Bohemia, New York, USA) at maximum speed for 2 min. Beads were then pelleted by centrifugation at 21,000 × *g* for 1 min and the supernatants were transferred to a separate RNase-free tube, and then mixed with 650 µL of 100% ethanol. Protocol 7 in the Qiagen RNeasy Mini Kit Handbook was then followed. Samples were eluted in 50 µL of RNase-free water, and the concentration and purity of each RNA sample was quantified spectrophotometrically using a NanoDrop 8000 instrument (Thermo Scientific).

Contaminating gDNA was removed from RNA samples using the RapidOut DNA Removal Kit (Thermo Scientific, Vilnius, Lithuania) according to manufacturer's instructions. RNA integrity was subsequently assessed using the Agilent Bioanalyzer system (Agilent Technologies); samples with a RNA integrity number (RIN) value ≥ 5 and/or clearly distinct rRNA banding patterns with little degradation were considered of appropriate quality for reverse transcription. All RNA samples were stored at -80ºC immediately after purification.

Complementary DNA (cDNA) was generated from 500 ng of each RNA sample using the High-Capacity cDNA Reverse Transcription Kit (Applied Biosystems, Foster City, CA, USA) with random hexamers according to manufacturer's instructions. Prior to qPCR, cDNA samples were diluted 1:6 for target genes of interest (*tcdA*, *tcdB, cdtA, cdtB)* and further diluted to 1:200 for the *rrs* reference gene. Quantitative PCR (qPCR) was carried out in 15 µL reaction volumes containing 5 µL of diluted cDNA, 7.5

μL of PerfeCTa SYBR® Green SuperMix with ROX (Quantbio) and 500 nM of each forward and reverse primer **(Supplementary Table S2 online)** using the StepOnePlus Real-Time PCR System (Life Technologies). Thermocycling conditions were as follows: 95ºC for 33 s and 60ºC for 30 s, repeated for 40 cycles. PCR product specificity was determined by melt curve analysis generated by completing a stepwise gradient 60ºC to 95ºC at a rate of 0.3ºC per second at the end of the qPCR run.

A threshold of 0.5 was used to determine the $C_q$ value for each amplicon using the StepOnePlus Real-Time software (Life Technologies). Gene expression was normalized to the *C. difficile* reference gene *rrs,* using the $\Delta C_q$ method. Error bars represent the standard deviation of three biological replicate experiments run in technical triplicate. Normality of $\Delta C_q$ values were assessed at 12 and 24 h time points for each *C. difficile* ribotype strain using the D'Agostino & Pearson normality test. To determine the significance between treatments (defined microbial ecosystem spent-medium) of normally distributed data, a one-way ANOVA followed by Dunnett's multiple comparisons test was performed on $\Delta C_q$ values compared to the DEC58 treatment group.

## References:

Carter, G. P. *et al.* Binary toxin production in *Clostridium difficile* is regulated by CdtR, a LytTR family response regulator. *J Bacteriol.* **189**, 7290-7301(2007).

Deneve, C., Delomenie, C., Barc, M. C., Collignon, A. & Janoir, C. Antibiotics involved in *Clostridium difficile*-associated disease increase colonization factor gene expression. *J Med Microbiol.* **57**, 732-738 (2008).

Gloor, G. B. *et al.* Microbiome profiling by illumina sequencing of combinatorial sequence-tagged PCR products. *PLoS One.* **5**, e15406 (2010).

Metcalf, D. S. Molecular investigation of the *Clostridium difficile* binary toxin. PhD thesis, University of Guelph (2012).
